# Supplementary material for: Target product profiles for novel medicines to prevent and treat preeclampsia: An expert consensus
Source: PLOS Glob Public Health. 2022 Nov 18;2(11):e0001260. doi: 10.1371/journal.pgph.0001260 (PMC10021561; doi:10.1371/journal.pgph.0001260)
Supplement: S1 Appendix — (DOCX) [file pgph.0001260.s001.docx]

| Interviewer/s |  |
| --- | --- |
| Date |  |
| Start and finish time |  |
| Participant code |  |
| Participant field |  |

Preamble:

Thanks for agreeing to participate in this interview. For your information, all the comments you provide will be treated as non-identifiable, which means that we will not attribute these comments to you by name, nor will anything that could identify you be released outside of our study team. Any feedback you provide will be captured through field notes that we will take during this discussion. If there are any questions you do not feel comfortable responding to, you do not have to answer them. You are welcome to ask any questions of us before we start. We would also like to ask your permission to record this interview, so that we may refer back to it if needed. The recording will not be shared outside the research team and will be destroyed at the completion of the study.

Verbal consent to participate

Verbal consent to record interview

The purpose of this interview is to discuss your opinions on the two target product profiles that we have developed for new drugs to prevent and treat pre-eclampsia. To start I give you a brief overview of what target product profiles (or TPPs) are: TPPs identify upfront both the minimum and preferred characteristics a product should take, in order to fulfil a specific, unmet clinical and public health need. If I could take you through the TPP structure: the TPP includes a brief description of the problem, as well as the scenario in which new drugs would be used. This is followed by a large table that describes the various characteristics of the drug (such as the target population, the safety, the stability and shelf-life), and the minimum requirements new drugs would have to meet (including not limiting the innovation of new drug technologies), as well as the preferred (or optimistic) requirements.

**TPP1: Novel treatments for the prevention of pre-eclampsia**

We will start by discussing the TPP for drugs to prevent pre-eclampsia. Pre-eclampsia is a major cause of maternal mortality globally. Currently, there are only two drugs recommended for the prevention of pre-eclampsia, aspirin, which prevents ~10-20% of cases of pre-eclampsia in high risk women, and calcium supplementation in women who live in regional areas with low calcium intake. Despite these drugs, pre-eclampsia contributes a huge burden on maternal and newborn health world-wide.

| Q1: Reading the preamble, is the problem definition for this TPP clear? |
| --- |
|  |
| Q2: Do you agree or disagree on the definition given for the intended use case scenario? |
|  |
| Q3a: Jumping into the TPPs themselves, we would like to discuss ***(insert appropriate key variables for participants area of expertise)*** with you, given your expertise. Would you agree or disagree on the minimum and preferred characteristics for ***(specific variable)***? |
|  |
| Q3b: Are there any other variables on which you would like to share your thoughts?  *If yes*, Would you agree or disagree on the minimum and preferred characteristics for ***(specific variable)***? |
|  |
| Q4: Are there any domains that you were uncomfortable with? |
|  |
| Q5: Do you see any significant gaps in the TPP? |
|  |
| Q6: Do you think that this TPP is designed to be applicable to all countries and income settings? |
|  |
| Q7: Could you suggest improvements, both for the content and structure of the document? (Was the content clear and easy to digest?) |
|  |

**TPP2: Novel drugs to treat pre-eclampsia**

Now we are going to discuss our second TPP, for new drugs to treat pre-eclampsia. The key difference between the first TPP and this one is that now we focused on drugs to give to women who have pre-eclampsia, to reduce the morbidity and mortality associated with pre-eclampsia and prolong pregnancy. Despite the availability of drugs that can treat the symptoms of pre-eclampsia such as anti-hypertensives or magnesium sulfate, the only cure for pre-eclampsia is birth of the baby and delivery of the placenta.

| Q1: Reading the preamble, is the problem definition for this TPP clear? |
| --- |
|  |
| Q2: Do you agree or disagree on the definition given for the intended use case scenario? |
|  |
| Q3a: Jumping into the TPPs themselves, we would like to discuss ***(insert appropriate key variables for participants area of expertise)*** with you, given your expertise. Would you agree or disagree on the minimum and preferred characteristics for ***(specific variable)***? |
|  |
| Q3b: Are there any other variables on which you would like to share your thoughts?  *If yes*, Would you agree or disagree on the minimum and preferred characteristics for ***(specific variable)***? |
|  |
| Q4: Are there any domains that you were uncomfortable with? |
|  |
| Q5: Do you see any significant gaps in the TPP? |
|  |
| Q6: Do you think that this TPP is designed to be applicable to all countries and income settings? |
|  |
| Q7: Could you suggest improvements, both for the content and structure of the document? (Was the content clear and easy to digest?) |
|  |
| Q1: Reading the preamble, is the problem definition and target user group of this TPP clear? |
|  |
| Q2: What do you think of the intended use case scenario? |
|  |
| Q3: Jumping into the TPPs themselves, we would like to discuss ***(insert appropriate key variables for participants area of expertise)*** with you, given your expertise. Are there any other variables on which you would like to share your thoughts? |
|  |
| Q4: Are there any domains that you were uncomfortable with? |
|  |
| Q5: Do you see any significant gaps in the TPP? |
|  |
| Q6: Do you think that this TPP is designed to be applicable to all countries and income settings? |
|  |
| Q7: Could you suggest improvements, both for the content and structure of the document? (Was the content clear and easy to digest?) |
|  |
