# Supplementary material for: Target product profiles for novel medicines to prevent and treat preeclampsia: An expert consensus
Source: PLOS Glob Public Health. 2022 Nov 18;2(11):e0001260. doi: 10.1371/journal.pgph.0001260 (PMC10021561; doi:10.1371/journal.pgph.0001260)
Supplement: Attachment — Submitted filename: PGPH-D-22-01331 response to revieweres.docx [file pgph.0001260.s002.docx]

PGPH-D-22-01331 “Target product profiles for novel medicines to prevent and treat preeclampsia: an expert consensus”

Response to reviewers

To the Editors,

Thank you for considering our paper for publication in PLOS global Health and thank you to all reviewers for their comments. We have amended the manuscript following feedback from reviewers and have addressed all comments in detail below. We look forward to hearing your response.

Regards,

Dr Annie McDougall on behalf of all authors.

Reviewer #1

We thank the reviewer for the feedback on the manuscript and have amended the manuscript to address all comments, providing specific feedback below.

*Introduction*

1. *It would strengthen their rationale for developing the TPP if they point to other global efforts, not just WHO, even though they may be outside of direct obstetric care.*

We have amended the language and references to include UNICEF target product profiles, lines 117 – 121.

*Methods*

2. *The use-case scenarios seem to be inconsistent in that the treatment scenario identified who will provide the drug (i.e., skilled health personnel) and the prevention scenario only says that the drug will be administered to pregnant women. Why was there not standardization of the two scenarios?*

We thank the reviewer for highlighting this. The inclusion in the use-case scenario for who will provide the drug was deliberately left out of the TPP for medicines to prevent PE, due to the broad nature of this variable in the TPP. Women taking medicines to prevent preeclampsia may be treated by skilled health personnel, especially if the medicines is administered invasively. However, they may also be self-administering the medicine at home, as is currently the case with aspirin. This is detailed in the TPP under the variable heading “Primary Target Delivery Channels”. We felt this was too complex to try and insert all these different scenarios into the use-case scenario for prevention.

Conversely, women presenting with preeclampsia and needing treatment will be treated in a community or healthcare facility and as such would be treated by a skilled health personnel.

3. *Citations should be provided for the "templates and guidance produced by several reputable organizations (FDA, Gates Foundation, WHO, PATH and others)"*

This has been added, line 163

4. *In step 2, 39 stakeholders were invited to participate. Please provide further information about the eligibility criteria for participation, and the sample size (n=39).*

This has been added, lines 199 – 204.

5. *[Add dates here] needs to be corrected*

This has been corrected, line 232

6. *Please explain Qualtrics. "The survey was conducted using Qualtrics and...".*

This has been added, lines 172-173

7. *Survey invitations were sent to approximately 270 individuals using several databases however the databases used were highly skewed towards previous participation in some kind of WHO activity. I have been working explicitly in the PE/E field for several years and never heard about this "international survey" so it makes me wonder how far-reaching the invitation really was. For example, was the invitation disseminated via the RHSC Maternal Health Caucus and similar implementing agency channels?*

In addition to identification of stakeholders via previous participation in WHO maternal and newborn health activities, our survey was disseminated via the listservs of the Cochrane Pregnancy and Childbirth network and the Perinatal Society of Australia and New Zealand.

Participation in WHO activities also delivers a wide and diverse group of stakeholders. For example, there was overlap identified between members of the RHSC and WHO activity participation. We had two interview participants on from the RHSC maternal health supplies caucus.

Our results demonstrate that we had diversity of geographical, gender and expertise from survey participants. Respondents were across all WHO geographical regions (*AFR* 19.6%, *AMR* 27.5%, *EUR* 15.7%, *SEAR* 11.8%, *WPR* 23.5%, *EMR* 2.0%). Different professions were represented, including researchers (44.7%), clinicians (doctor, midwife or nurse, 23.4%), epidemiologists or public health specialists (10.6%), staff or consultants of a national or international NGOs (6.4%), staff of funding agencies (6.4%), employee or consultants of a normative body or civil society organisation (2.1%) or other (including educator, research student and combined clinician/researchers, 6.4%).

8. *Invitation for public consultation was disseminated via the Burnet Institute Twitter account. How many responses were captured via this "public consultation"? This seems to be a rather nominal way to obtain public input. For this reason, it would be helpful to understand the extent of "public" comment. Why was this method selected as a way to gain public comment?*

Unfortunately, only one response was received via the public consultation period. The feedback gained from this one response was in accordance with the large volume of feedback received from the interviews and surveys. This method of public comment was chosen as it is a similar method prescribed in the WHO standard operating procedure for public comment on TPPs, which propose that public comment from the WHO website be available for 4-8 weeks. We conducted our public comment consultation via the Burnet website, with Burnet social media channels being used to draw people’s attention to the call for comments.

*Results*

9. *Throughout this section, it would be helpful to put sample sizes where results are being reported. Also, it would be helpful to keep the order the same as when it was described in the methods section. In the current manuscript, it is difficult to track the various data collection efforts so some clarity is required.*

We have added the sample size to the legend for Figure 2, line 342. Sample size is already presented in the results (line 308 and 323). We feel it is inappropriate to provide sample sizes thoughout the presentation of the results from the interviews as these were qualitative results.

We have altered the order of the methods section, so that it is presented in the same order as the results (lines 168 – 193)

10. *Figure 2 should be labeled better; what is A, B, C D? The narrative that describes Figure 2 is confusing.*

We have altered the language to make the distinction between the panels in this figure easier to understand. Lines 345 – 346

11. *I think it would be easier to follow this discussion if the final TPPs for both prevention and treatment (Tables 1 and 2) were presented first since the narrative related directly to the TPPs.*

We have made this suggested amendment.

*Discussion*

12. *PE/E diagnostics have already benefitted from TPPs for specific product development efforts Thank you for pointing out this error. It has been amended (Table 2).*

We thank the reviewer for this suggested reference. Although there is a Devices, Diagnostics, and Drugs to Address Women’s Needs Product Development Partnership (D3AWN PDP) headed by PATH, there is no publically available TPP for preeclampsia diagnostics. This reference describes the advancement of a specific diagnostic test for preeclampsia and includes a use-case and go-to-market plan. We have included this as a reference for the sentence “Preeclampsia diagnostics may themselves be a strong candidate for new TPPs in the future”. Line 609

13. *I am not sure many people know what "triallists" are? Perhaps rephrase?*

This has been amended to “researchers conducting clinical trials”, lines 680-681.
